# The correlation of fibrinogen-like protein-1 expression with the progression and prognosis of hepatocellular carcinoma

**DOI:** 10.1007/s11033-022-07624-6

**Published:** 2022-07-01

**Authors:** Nanni Hua, Anxian Chen, Chen Yang, Hui Dong, Xianglei He, Guoqing Ru, Xiangmin Tong, Feifei Zhou, Shibing Wang

**Affiliations:** 1 The Second Clinical Medical College, Zhejiang Chinese Medical University, Hangzhou, 310000 China; 2 Cancer Center, Molecular Diagnosis Laboratory, Key Laboratory of Tumor Molecular Diagnosis and Individualized Medicine of Zhejiang Province, Zhejiang Provincial People’s Hospital, Affiliated People’s Hospital, Hangzhou Medical College, Hangzhou, 310014 Zhejiang People’s Republic of China; 3 School of Public Health (Shenzhen), Shenzhen Campus of Sun Yat-Sen University, Shenzhen, 518107 Guangdong China; 4 Department of Ultrasound, Zhejiang Provincial People’s Hospital, Affiliated People’s Hospital, Hangzhou Medical College, Hangzhou, 310014 Zhejiang China; 5 Department of Stomatology, Bengbu Medical College, 2600 Donghai Avenue, Bengbu, 233030 China; 6 Departments of Pathology, Zhejiang Provincial People’s Hospital, People’s Hospital of Hangzhou Medical College, Hangzhou, 310014 Zhejiang China; 7 Departments of TCM Gynecology, Zhejiang Provincial People’s Hospital, People’s Hospital of Hangzhou Medical College, Hangzhou, China

**Keywords:** Fibrinogen-like-protein 1, Hepatocellular carcinoma, Progression, Prognosis, Tumor suppressor

## Abstract

**Background:**

Fibrinogen-like-protein 1 (FGL1), a member of the fibrinogen-related protein (FREP) family, is a major ligand of the immune inhibitory receptor lymphocyte-activation gene 3 (LAG-3). While FGL1 is strongly implicated in the development and prognosis of a variety of diseases, its role in hepatocellular carcinoma (HCC) is still disputed. Therefore, the role of FGL1 expression in the progression and prognosis of HCC was investigated.

**Methods and results:**

In the present study, bioinformatics analysis was first used to probe the expression profile of FGL1 in multiple malignant tumor tissues and paired normal tissues, and to explore the possible relationship between FGL1 and prognosis of HCC patients. Thereafter, the expression levels of FGL1 were determined and compared in human HCC cell lines, HCC tissues, peri-tumor tissues and normal liver tissues by western blot analysis. Furthermore, tissue microarrays were used to detect the expression of FGL1 through immunohistochemical staining and to verify whether the FGL1 expression level was associated with clinicopathological features and the prognosis of HCC patients. The results showed that FGL1 was downregulated significantly in most of the HCC cells lines and HCC tissues, corresponding to the results of the bioinformatics and western blot analyses. FGL1 expression level in HCC was found to be correlated to Edmondson grade and metastasis of the HCC. Additionally, high FGL1 expression was associated with better overall survival in HCC patients, suggesting that FGL1 could function as a tumor suppressor.

**Conclusions:**

The expression level of FGL1 can be correlated with the progression and prognosis of HCC, suggesting its potential as a prognostic biomarker.

## Introduction

According to 2020 data, hepatocellular carcinoma (HCC) is the sixth most commonly diagnosed and the third most deadly malignant tumor in the world, with 905,677 new cases and 830,180 reported deaths [1]. Due to the insidious onset of HCC and the limited availability of targeted drugs, the five-year survival rate of HCC patients in China is only 14.1% [2]. Reliable prognostic indicators could improve the situation, but the sensitivity and specificity of current biomarkers are inadequate [3, 4]. Therefore, it is critical to seek effective biomarkers which can predict the prognosis and guide the treatment for HCC to further improve clinical outcomes.

Fibrinogen-like-protein 1 (FGL1), also known as hepassocin or hepatocyte-derived fibrinogen-related protein 1 (HFREP1), is a liver-secreted protein with two disulfide-linked 34kD homodimers [5, 6]. FGL1 was first found to be over-expressed in HCC [7, 8]. As a member of fibrinogen-related protein (FREP) family, FGL1 is closely correlated with the development and prognosis of a variety of diseases. Moreover, a previous study suggested that FGL1 can activate mitosis, increase metabolic activity, and is closely related to obesity [9]. Under normal physiological conditions, FGL1 is involved in fine-tuning systemic inflammation by facilitating an interaction between the liver and other peripheral tissues [10]. FGL1 can also stimulate the EGFR/ERK cascade to promote hepatocyte proliferation via the Src-dependent pathway [11, 12]. In addition, FGL1 acts as a liver regeneration factor with previously reported increase in expression during the liver regeneration [13–15]. In summary, FGL1 has a vital effect on liver regeneration and protection. However, FGL1 expression not only affects hepatocyte regeneration but also regulates the growth and proliferation of tumor cells [16, 17]. Targeted disruption of FGL1 has been shown to accelerate the development of HCC, which suggests that FGL1 could be a potential therapeutic target in HCC patients [17]. Another study recently found that FGL1 is an important component of the FGL1-LAG-3 pathway that promotes the growth of malignant tumors, suggesting that the double blockade of FGL1 and PD-1/PD-L1 could become a therapeutic alternative for patients for whom anti-PD therapy is ineffective [18]. Interestingly, while FGL1 is downregulated in HCC compared to normal tissues, it has been found to be upregulated in melanoma, lung, breast, and colorectal cancers [6]. Overall, the role of FGL1 in HCC remains controversial and warrants further investigation.

The present study employs bioinformatics analysis to probe the expression profile of FGL1 in multiple malignant tumor tissues and paired normal tissues, and to explore the possible relationship between FGL1 and HCC prognosis. Moreover, western blot was used to further compare the expression levels of FGL1 in HCC cell lines, HCC tissues, peri-tumor tissues and normal liver tissues. Furthermore, a large sample size was used to determine whether FGL1 expression level is associated with the clinicopathological features and prognosis of HCC. The results of the present study shed light on a possible prognostic biomarker which could help in the development of new treatments against HCC as well as in the evaluation of HCC prognosis.

## Materials and methods

### Patient population and tissue samples

Samples from 237 HCC patients, admitted in the Zhejiang Provincial People’s Hospital between January 1998 to December 2011, were included in the study. Samples from tumor, peri-tumor, and normal liver tissues were collected from the participants, in addition to their clinical information (including data about age, gender, tumor size, tumor number, Edmondson grade, metastasis, microvascular invasion, hepatitis B surface antigen [HBsAg], cirrhosis, alpha fetoprotein [AFP] and so on). All participants were followed up for more than 5 years from the time of their surgical operation until December 2018 or patient death. All participants were in the 25–90 years age group, with an average age of 57.5 years. Of the 237 participants, 47 (19.8%) were women and 190 (80.2%) men. At initial diagnosis, 59.2% of the participants had HCC tumors less than or equal to 5 cm in diameter, while 40.8% patients had tumors greater than 5 cm in diameter. Nearly 65.2% of the HCC patients suffered from Edmondson grade I/II disease, while 34.8% suffered from grade III HCC. The numbers of patients with and without metastasis were 19 (8.3%) and 210 (91.7%), respectively. The study was authorized by the Ethics Committee of the Zhejiang Provincial People’s Hospital (2019KY232). Importantly, informed consent was obtained from all study participants.

### Cell culture

Human HCC cell lines (HCC-LM3, SK-Hep1, SMMC-7721, SNU182, C3A, HepaG2, Huh7, Hep3B) and human normal liver cell L02 were purchased from the ATCC. All cell lines were cultured in Dulbecco’s modified Eagle medium (DMEM, Gibco, USA) with 10% fetal bovine serum (Gibco, USA), 100 units/mL penicillin, and 100 µg/mL streptomycin (Life Technologies, USA) under 5% CO_2_ at 37 °C.

### Western blot assay

Total proteins were extracted from the HCC tissue samples and cell lines in RIPA buffer supplemented with a protease inhibitor cocktail (Roche, Switzerland). A BCA kit (Beyotime, China) was used to measure the protein concentration. The protein samples were boiled at 100 °C for 5 min, and separated using 12% SDS-PAGE. The separated proteins were electro transferred onto 0.22 μm PVDF membranes (Roche, Switzerland), and sealed with 5% skim milk for 1 h. Next, the membranes were incubated with anti-GAPDH (Bioworld, USA, 1:2000 diluted) or anti-FGL1 (ab197357, Abcam, England,1:1000 diluted) primary antibody at 4 °C overnight, followed by washing in tris-buffered saline with 0.1% (v/v) Tween® 20 (TBST) for 3 × 10 min. Subsequently, the membranes were incubated with the secondary antibody (1:2000 diluted) for 1 h, followed by washing in TBST for 3 × 10 min. Thereafter, the FDbio-Dura enhanced chemiluminescence (ECL) reagent (FD8020, FDbio science, China) was used to detect signals under the Alpha Innotech Fluor Chem-FC2 imaging system (Protein Simple, USA). GAPDH was used as an internal control.

### Bioinformatics analysis of FGL1 expression in HCC

The Gene Expression Profiling Interactive Analysis 2 (GEPIA 2) (http://gepia2.cancer-pku.cn/#index) online website was used to analyze the FGL1 levels in malignant tumors. Firstly, the dot plot gene expression profile of FGL1 was obtained by performing a single gene analysis across multiple malignant tumor tissues and paired normal tissues. Secondly, expression DIY was used to analyze FGL1 expression in HCC with a box plot, setting the parameters as follows: gene; Gene A, FGL1; [Log_2_FC] Cutoff, 1; p-value Cutoff, 0.01; Multiple Datasets; Datasets Selection (Cancer name), LIHC; Log Scale, Yes; Jitter Size, 0.4; Match Normal data, Match TCGA normal and GTEx data. Thereafter, a possible relationship between FGL1 and the clinical stage of HCC was explored by using the UALCAN online website (http://ualcan.path.uab.edu/). Finally, a Kaplan–Meier curve of overall survival (OS) was plotted using GraphPad Prism 8.0 on the basis of the OncoLnc Database (http://www.oncolnc.org/).

### Immunohistochemistry staining

Tissue specimens, including HCC tissues and adjacent normal liver tissues, were fixed with formalin and embedded in paraffin. The 5-µm-thin paraffin-embedded tissue microarray (TMA) sections subsequently obtained were dewaxed in xylene (Sinopharm, China) and serially rehydrated in alcohol solutions (Sinopharm, China). Thereafter, the tissue sections were incubated with 3% hydrogen peroxide (Sinopharm, China) in order to block endogenous peroxidases. To reduce nonspecific protein binding, the sections were subsequently incubated in 1% (w/v) bovine serum albumin (BSA; Sigma, Germany) for 20 min. The sections were then incubated with an anti-FGL1 polyclonal antibody (1:50; HuaBio, Hangzhou, China) at 25 °C for 1 h, followed by incubation with a biotinylated secondary antibody (MXB, Fuzhou, China) at 37 °C for 30 min. Thereafter, the TMA sections were stained with 3,3′-diaminobenzidine (DAB) chromogen (Gene Tech, Shanghai, China), followed by counterstaining with Mayer’s hematoxylin (HuaBio, Hangzhou, China). Finally, all tissue sections were destained with alcohol and xylene. The sections were observed using an inverted fluorescence microscope. The immunohistochemical staining of FGL1 was scored into four grades, judged independently by two pathologists, based on the intensity and proportion of the positively stained cells. In the present study, grade 1 was assigned for low FGL1 expression, followed by higher grades for higher FGL1 expression.

### Statistical methods

SPSS 25.0 (Chicago, IL, USA) was used to analyze all data. An independence test of categorical variables was performed using chi-square analysis or Fisher’s exact test. In addition, the Kaplan–Meier analysis was performed to evaluate any differences in survival. Variables with *P* < 0.1 in the univariate analysis were incorporated into the proportional hazard model of Cox for multivariable analysis. Differences were deemed statistically significant at *P* < 0.05.

## Results

### Expression profile of FGL1 on GEPIA 2 and its relation with HCC prognosis

**Fig. 1:**
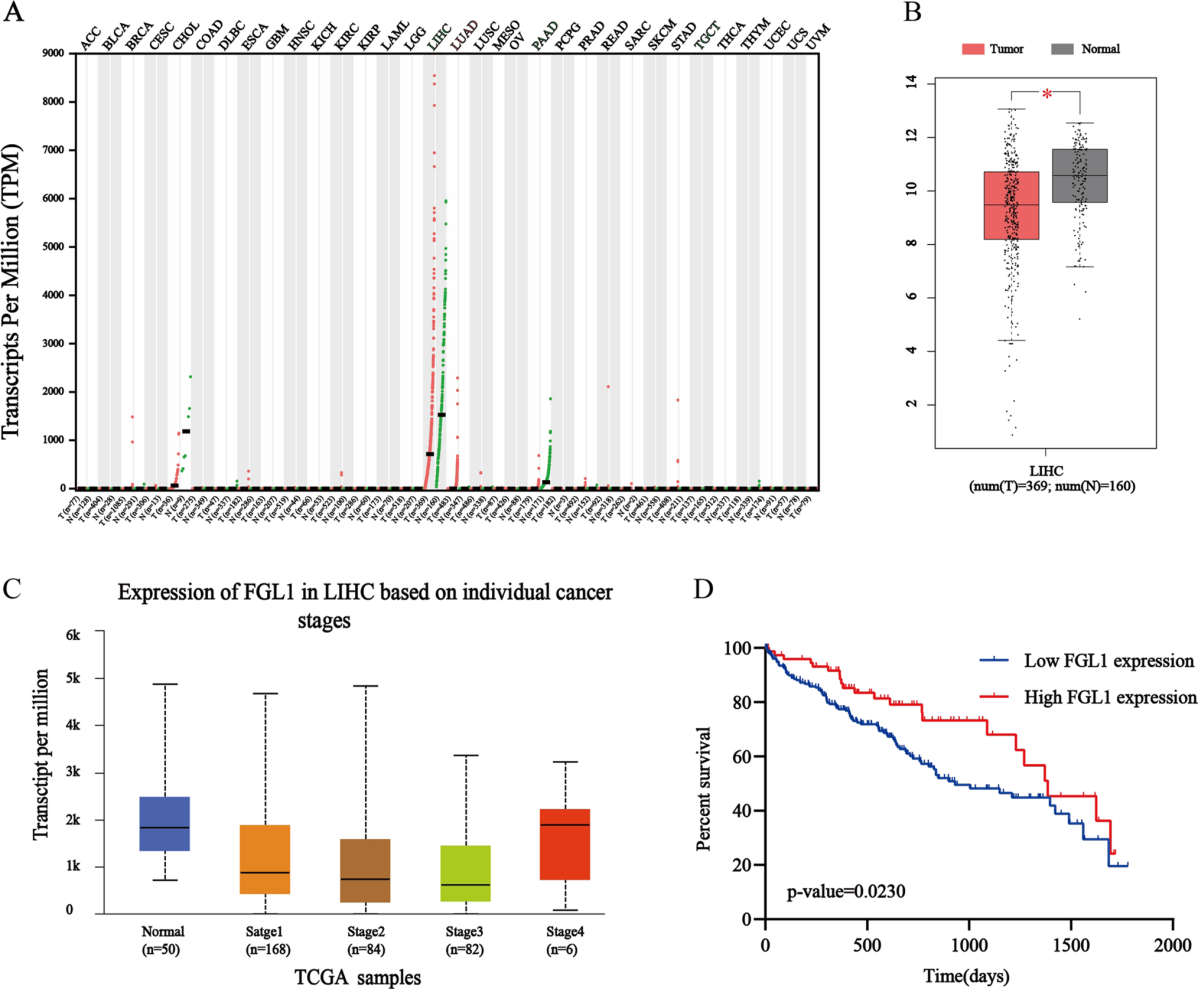
FGL1 expression of HCC tissues was downregulated compared with paired normal tissues, and the downregulation of FGL1 was correlated with poor HCC prognosis. **A** FGL1 expression profile across all cancer samples and paired normal tissues on the basis of GEPIA. **B** The differential expression level of FGL1 in tumor and non-tumorous liver tissues. **C** FGL1 expression in LIHC based on individual pathological stages by UALCAN online website. **D** Kaplan–Meier survival curves of LIHC patients with low and high FGL1 expression based on the OncoLnc Database. “*” signifies *P* value < 0.05

The expression profile of FGL1 in multiple malignant tumors was bioinformatically determined using the GEPIA 2 webserver. Expectedly, the expression of FGL1 in the liver hepatocellular carcinoma (LIHC) was higher than in any other malignant tumor tissues, and lower than in normal liver tissues (Fig. 1A). There was a significant difference in the expression of FGL1 between normal liver tissues and LIHC (Fig. 1B, *P* < 0.05) (matched TCGA normal and GTEx data). Further, a search of existing OMICS data using the UALCAN website suggested that FGL1 expression was significantly related to individual cancer stages (Fig. 1C, *P* < 0.05).

Based on the above results, it was speculated that FGL1 could be correlated to the prognosis of HCC patients. To further explore this correlation, the OncoLnc database was used to conduct a survival analysis based on the expression status of FGL1. The FGL1 expression level was found to be associated with the prognosis of HCC patients, with low FGL1 expression indicating poor overall survival (OS) (Fig. 1D, *P* = 0.023).

### Evaluation of FGL1 expression in human HCC cell lines and HCC tissues

**Fig. 2:**
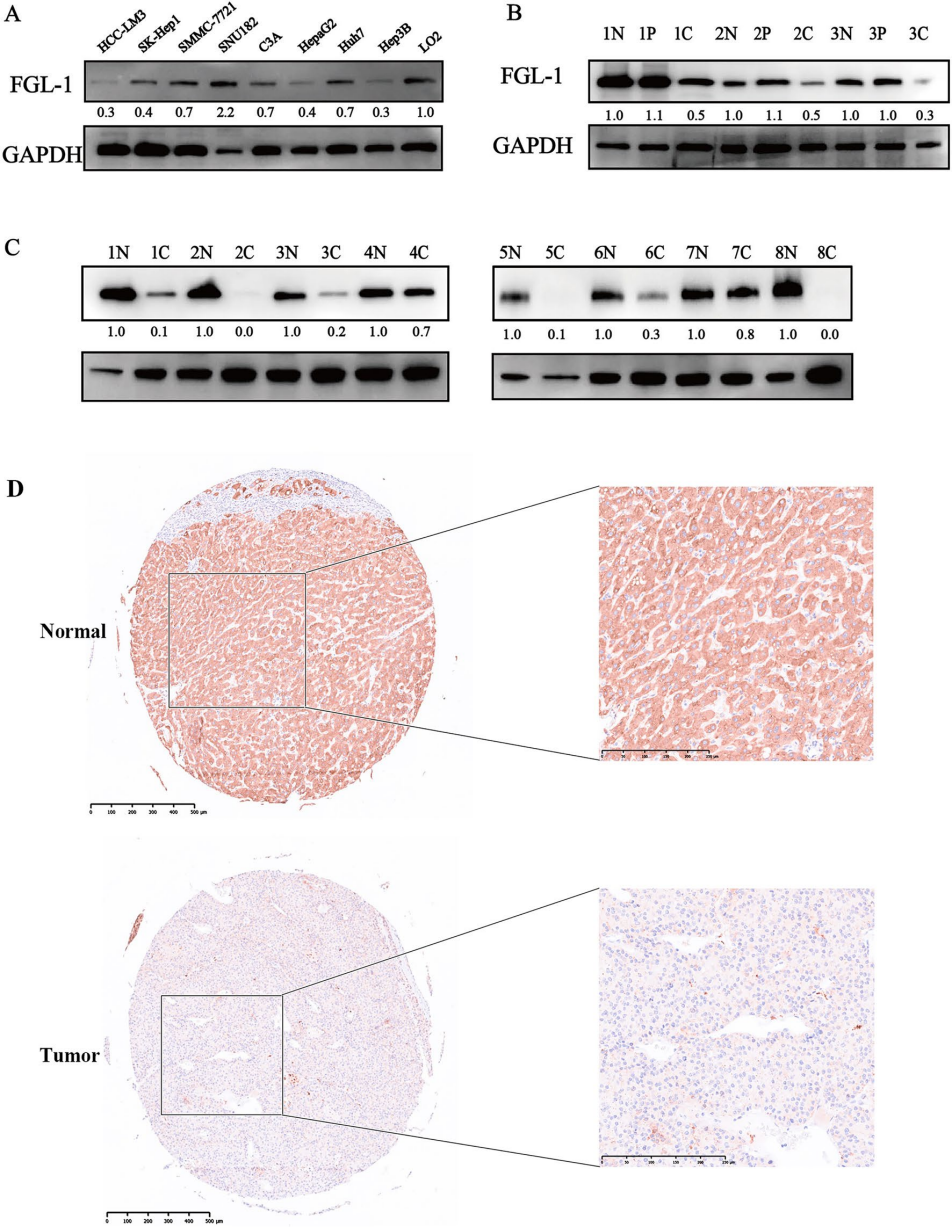
FGL1 expression showed obvious downregulation in several human HCC cell lines and HCC tissues. **A** Determination of FGL1 expression in several human HCC cell lines and the normal liver cell line via western blot analysis. **B** Determination of FGL1 expression with 3 pairs of HCC tissues and peri-tumor tissues and paired normal liver tissues via western blot analysis. **C** Determination of FGL1 expression with 8 pairs of HCC tissues and paired normal liver tissues via western blot analysis. **D** IHC staining for tumor tissues and adjacent normal liver tissues from HCC patients in the TMA. *N* normal liver tissue, *P* peri-tumor tissue, *C* cancer tissue; the numbers before N, *P* and C represent the group number

To further explore the positive results of bioinformatics analysis, FGL1 expression was tested in human HCC cell lines and HCC tissues. The expression profile of FGL1 in human HCC cell lines was determined by western blotting. A quantitative comparison, performed using the gray analysis of the western blot, revealed a lower FGL1 expression in the HCC-LM3, SK-Hep1, SMMC-7721, C3A, HepaG2, Huh7, and Hep3B cells, compared to the normal liver cell line (LO2) (Fig. 2A). Further, the FGL1 expression profile for 11 pairs of fresh tissues collected from HCC patients revealed that the expression was the lowest in HCC tissues, compared to normal and peri-tumor tissues (Fig. 2B). Similarly, as expected, FGL1 expression was lower in HCC tissues than in normal liver tissues (Fig. 2C). Notably, the IHC staining of TMAs also indicated that FGL1 expression was lower in HCC tissues compared to adjacent normal liver tissues (Fig. 2D). Overall, the above results confirmed that FGL1 expression in HCC tissues is lower than normal tissues, which also corroborates with the trend observed in the bioinformatics analysis.

### Correlation analysis between FGL1 expression and the clinicopathological parameters of HCC

Based on the four grades of FGL1 expression, all patients were divided into high and low expression groups to probe the correlation between FGL1 expression levels and the clinicopathological parameters of HCC. FGL1 expression levels in HCC were found to be correlated with Edmondson grade and metastasis (Table1, *P* < 0.05). However, no significant correlation of FGL1 expression was found with other clinical parameters (age, gender, tumor size, tumor number, microvascular invasion, HBsAg, cirrhosis, and AFP) (Table1, *P* > 0.05).

**Table 1:** Expression of FGL1 in hepatocellular carcinoma tissues

Clinical parameters	Number	FGL1 expression		χ^2^	*P* value
		Low	High		
Age (years)				0.012	0.913
< 55	90	13	77		
≥ 55	147	22	125		
Gender				0.894	0.344
Male	190	26	164		
Female	47	9	38		
Tumor size				0.651	0.420
≤ 50 mm	138	18	120		
> 50 mm	95	16	79		
Tumor number				0.095	0.758
Single	194	28	166		
Multiple	43	7	36		
Edmondson grade				4.069	0.044*
I + II	146	17	129		
III	78	17	61		
Metastasis				8.453	0.004*
M0	210	26	184		
M1	19	7	12		
Microvascular invasion				0.670	0.413
Absence	87	11	76		
Presence	88	15	73		
HBs antigen				0.015	0.904
Negative	46	7	39		
Positive	186	27	159		
Cirrhosis				3.049	0.081
Negative	78	16	62		
Positive	159	19	140		
AFP(µg/L)				0.297	0.586
< 50	104	14	90		
≥ 50	86	14	72		

Total number was less than 237 due to incomplete pathological data

*Signifies *P* value < 0.05

### Prognostic significance of FGL1 expression level for HCC

**Fig. 3:**
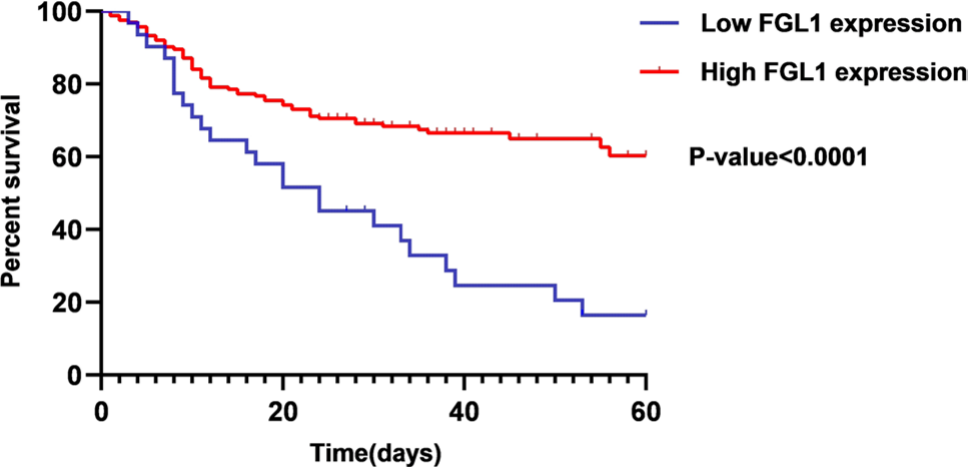
Kaplan–Meier survival analysis of HCC patients with different expression levels of FGL1 in TMA

The statistical significance of various prospective HCC prognostic factors was determined using the Cox regression analysis. Based on a univariate Cox regression analysis, tumor number, Edmondson grade, metastasis, HBsAg and FGL1 expression level could all be prognostic factors for HCC (Table 2, *P* < 0.1). A multivariate Cox regression analysis was performed to further ensure that Edmondson grade, metastasis and FGL1 expression level were independent prognostic factors for HCC (Table 2, *P* < 0.05). Consistently, it was found that FGL1 expression significantly correlated with OS in HCC patients on the Kaplan–Meier survival curve, with low FGL1 expression being linked to a shorter OS (Fig. 3, *P* < 0.0001).

**Table 2:** Univariate and Multivariate Cox regression of the clinicopathological parameters in HCC patients

Parameters	Univariate analysis				Multivariate analysis			
	Coefficient	HR	95.0% Cl For HR	*P*	Coefficient	HR	95.0% Cl For HR	*P*
Age (< 55 years/≥ 55 years)	− 0.295	0.744	0.370–1.498	0.408				
Gender (Male/Female)	− 0.199	0.819	0.370–1.814	0.623				
Tumor size (≤ 50 mm/> 50 mm)	0.376	1.457	0.659–3.218	0.352				
Tumor number (Single/multiple)	1.321	3.748	1.634–8.600	0.002*	0.519	1.680	0.941–2.998	0.079
Edmondson grade (I + II/III)	1.276	3.582	1.650–7.779	0.001*	1.076	2.932	1.751–4.909	0.000*
Metastasis (M0/M1)	0.842	2.320	0.894–6.023	0.084	1.402	4.062	2.139–7.712	0.000*
Microvascular invasion (−/+)	0.062	1.064	0.494–2.292	0.874				
HBs antigen (−/+)	− 1.025	0.359	0.127–1. 014	0.053	-0.033	0.968	0.532–1.760	0.915
Cirrhosis (−/+)	0.599	1.820	0.823–4.021	0.139				
AFP (< 50 µg/L / ≥ 50 µg/L)	0.251	1.285	0.598–2.762	0.520				
FGL1 (−/+)	− 0.924	0.397	0.171–0.922	0.032*	-0.861	0.423	0.247–0.725	0.002*

*Signifies *P* value < 0.05

## Discussion

While the diagnosis and treatment of HCC have improved in recent years, the prognosis of HCC patients is still poor. Various prognostic markers, such as AFP and AFU, have been employed extensively, but the specificity and sensitivity of these biomarkers are not sufficient [19]. The occurrence of false positives makes it challenging to differentiate early-stage HCC from other liver disorders such as acute hepatitis and cirrhosis [20]. Therefore, it is important to search for more convenient and more reliable markers that can facilitate early diagnosis and indicate the prognosis of HCC.

FGL1 is both a hepatic protectant and a hepatocyte mitogen involved in mitogenic and metabolic activity [21]. In the case of liver injury or acute inflammation, FGL1 expression levels increase [10, 15, 22], promoting the proliferation of normal hepatocytes in vivo. Similarly, FGL1 can also affect the proliferation of HCC cells. Downregulation of FGL1 in HCC cells may contribute to their growth and proliferation [23]. Further, FGL1 promotes hepatic cell proliferation by an autocrine mechanism, while inhibiting HCC cell proliferation via an intracrine pathway [24]. However, the exact role of FGL1 in HCC remains controversial. The present study was conducted to elucidate this role.

Previous studies have reported the downregulation of FGL1 in HCC, and a strong correlation between FGL1 expression and the differentiation status of malignant tumors [25]. The present study confirmed, using GEPIA 2, that FGL1 expression in HCC tissues is lower compared to normal liver tissues. Western blot analysis also confirmed that FGL1 expression is lower in most HCC cell lines (HCC-LM3, SK-Hep1, SMMC-7721, C3A, HepaG2, Huh7,and Hep3B cells) than normal liver cells (LO2). Furthermore, FGL1 expression in primary HCC tissues was also found to be lower than normal tissues and peri-tumor tissues. Moreover, FGL1 expression was significantly related to individual cancer stages, as determined through the UALCAN website. TMA results further demonstrated that FGL1 expression levels in HCC are correlated with the HCC’s Edmondson grade and metastasis. The significance of FGL1 as a prognostic marker for HCC was further confirmed using a survival analysis, which showed that FGL1 expression level is significantly correlated with OS in HCC patients (*P* < 0.05). Particularly, downregulation of FGL1 hints at poor OS. Multivariate Cox regression analysis confirmed that FGL1 expression level could be considered as a prognostic factor for HCC patients. Based on these data, it can be speculated that the FGL1 is linked to the progression and prognosis of HCC by functioning as a tumor suppressor.

Recent studies have increasingly focused on FGL1 for its possible role in the prognosis and treatment of various malignant tumors. In LKB1 mutant lung adenocarcinoma, loss of FGL1 was found to promote angiogenesis and epithelial-mesenchymal transition, leading to poor prognosis [26]. However, Yang et al. [16] reported that FGL1 upregulation is linked to poor prognosis of gastric cancer, contrary to the results of the present study in the context of HCC. The studies discussed above prove that FGL1 is associated with the prognosis of malignant tumors, and that it also plays a significant role in their treatment. A previous study has shown that the suppression of FGL1 inhibits the expression of caspase 3 and PARP1, thereby enhancing the inhibitory and apoptosis-inducing activities of gefitinib in the non-small cell lung cancer (NSCLC) cell line PC9/GR [27]. Similarly, Son et al. [28] reported that sorafenib-induced anti-tumor effects were enhanced by knocking down FGL1. Interestingly, high expression levels of FGL1 are related to high densities of LAG-3^+^cells, confirming that FGL1 is a high-affinity ligand for LAG-3 [29]. It is also known that blocking the FGL1-LAG-3 pathway can enhance the activation of T cells and promote anti-tumor immunity [18]. Hence, targeting the FGL1-LAG-3 pathway, in addition to anti-PD1 therapy, could significantly improve the treatment of HCC patients for whom anti-PD1 therapy alone is not effective [30]. Notably, it has been found that the drug oxysophocarpine decreases FGL1 expression by downregulating IL-6-mediated JAK2/STAT3 signaling, ultimately enhancing the immunotherapeutic effect of CD8^+^ T cells against HCC in vivo and in vitro [31]. In the future, the treatment and prognosis of various malignant tumors could be improved by adjusting the expression of FGL1 through such drugs.

The present study is reinforced with clinical data, providing strong evidence for the prognostic significance of FGL1 for HCC. Nevertheless, the underlying mechanisms of the signaling pathways in HCC remain unclear. Future studies would continue to explore the detailed mechanisms that can explain the correlation between FGL1 and HCC.

## Conclusions

The present study found that FGL1 expression is correlated with the progression and prognosis of HCC, suggesting that FGL1 could be a potential prognostic biomarker for HCC. Importantly, this study also provides a basis for further investigations of FGL1 in the context of HCC.

## Data Availability

The datasets supporting the conclusions of this article are included within the article.

## Abbreviations

AFP: Alpha fetoprotein
ECL: Enhanced chemiluminescence
FGL1: Fibrinogen-like-protein 1
FREP: Fibrinogen-related protein
GAPDH: Glyceraldehyde-3-phosphate dehydrogenase
GEPIA: Gene expression profiling interactive analysis
HBSAg: Hepatitis B surfaceantigen
HCC: Hepatocellular carcinoma
HFREP1: Hepatocyte-derived fibrinogen-related protein 1
IHC: Immunohistochemistry
LAG-3: Lymphocyte activation gene 3
LIHC: Liver hepatocellular carcinoma
LKB1: Liver kinase B1
NSCLC: Non-small cell lung cancer
OS: Overall survival
PVDF: Polyvinylidene difluoride
SDS-PAGE: Sodium dodecyl sulfate polyacrylamide gel electrophoresis
TBST: Tris buffered saline with tween
TCGA: The Cancer Genome Atlas
TMA: Tissue microarray
TPM: Transcripts per million
