# Correction: The correlation of fibrinogen‑like protein‑1 expression with the progression and prognosis of hepatocellular carcinoma

**DOI:** 10.1007/s11033-025-10805-8

**Published:** 2025-07-16

**Authors:** Nanni Hua, Anxian Chen, Chen Yang, Hui Dong, Xianglei He, Guoqing Ru, Xiangmin Tong, Feifei Zhou, Shibing Wang

**Affiliations:** 1 The Second Clinical Medical College, Zhejiang Chinese Medical University, Hangzhou, 310000 China; 2 Cancer Center, Molecular Diagnosis Laboratory, Key Laboratory of Tumor Molecular Diagnosis and Individualized Medicine of Zhejiang Province, Zhejiang Provincial People’s Hospital, Affiliated People’s Hospital, Hangzhou Medical College, Hangzhou, 310014 Zhejiang People’s Republic of China; 3 School of Public Health (Shenzhen), Shenzhen Campus of Sun Yat-Sen University, Shenzhen, 518107 Guangdong China; 4 Department of Ultrasound, Zhejiang Provincial People’s Hospital, Affiliated People’s Hospital, Hangzhou Medical College, Hangzhou, 310014 Zhejiang China; 5 Department of Stomatology, Bengbu Medical College, 2600 Donghai Avenue, Bengbu, 233030 China; 6 Departments of Pathology, Zhejiang Provincial People’s Hospital, People’s Hospital of Hangzhou Medical College, Hangzhou, 310014 Zhejiang China; 7 Departments of TCM Gynecology, Zhejiang Provincial People’s Hospital, People’s Hospital of Hangzhou Medical College, Hangzhou, China

**Correction to: Molecular Biology Reports (2022) 49:7911–7919**

10.1007/s11033-022-07624-6

In this article, Fig. 2D appeared incorrectly and has now been corrected in the original publication. For completeness and transparency, the correct and old incorrect versions are displayed below.

The original article has been corrected.

Incorrect version:

**Fig. 2:**
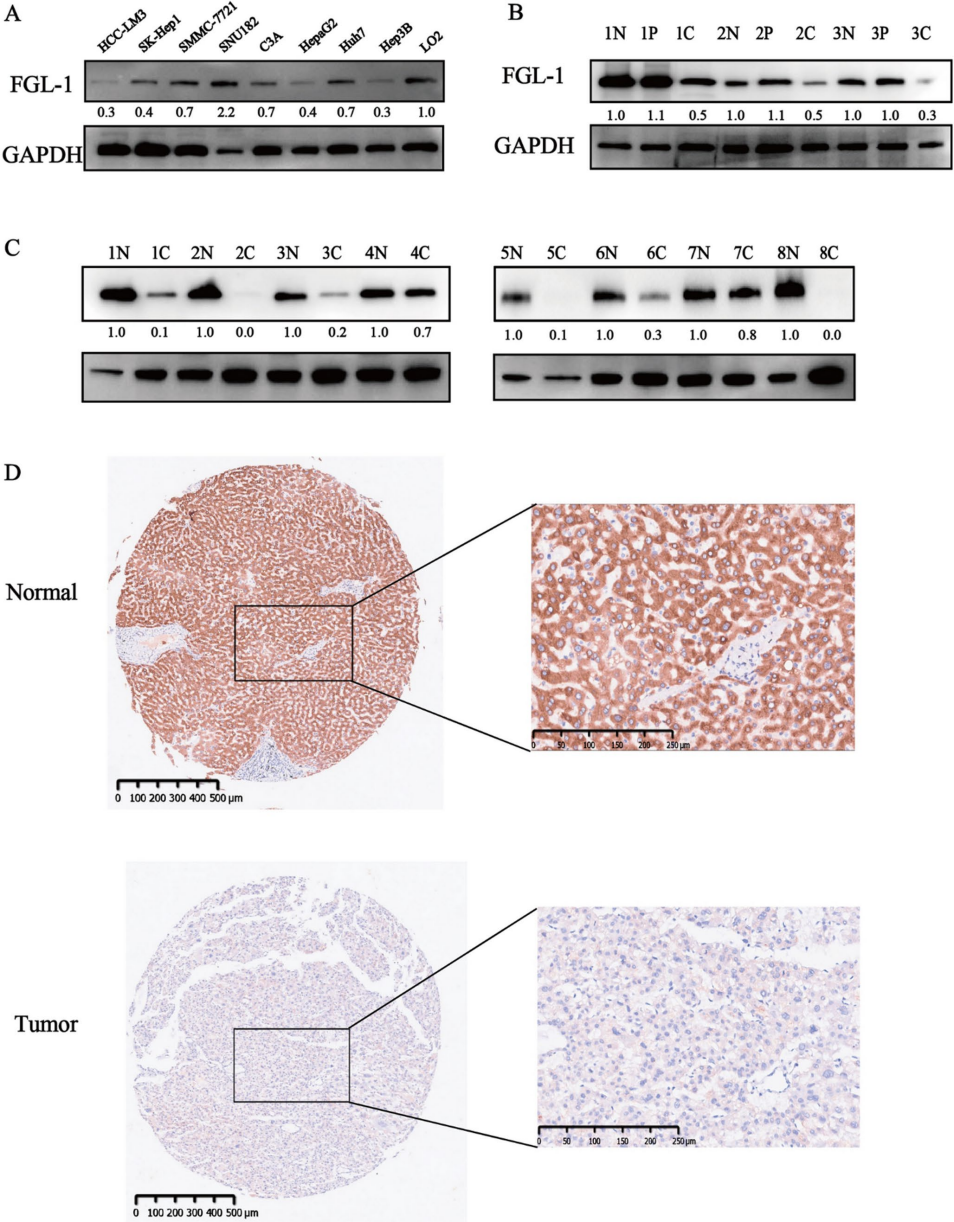
FGL1 expression showed obvious downregulation in several human HCC cell lines and HCC tissues. **A** Determination of FGL1 expression in several human HCC cell lines and the normal liver cell line via western blot analysis. **B** Determination of FGL1 expression with 3 pairs of HCC tissues and peri-tumor tissues and paired normal liver tissues via western blot analysis. **C** Determination of FGL1 expression with 8 pairs of HCC tissues and paired normal liver tissues via western blot analysis. **D** IHC staining for tumor tissues and adjacent normal liver tissues from HCC patients in the TMA. *N* normal liver tissue, *P* peri-tumor tissue, *C* cancer tissue; the numbers before N, *P* and C represent the group number

Correct version:

**Fig. 2:**
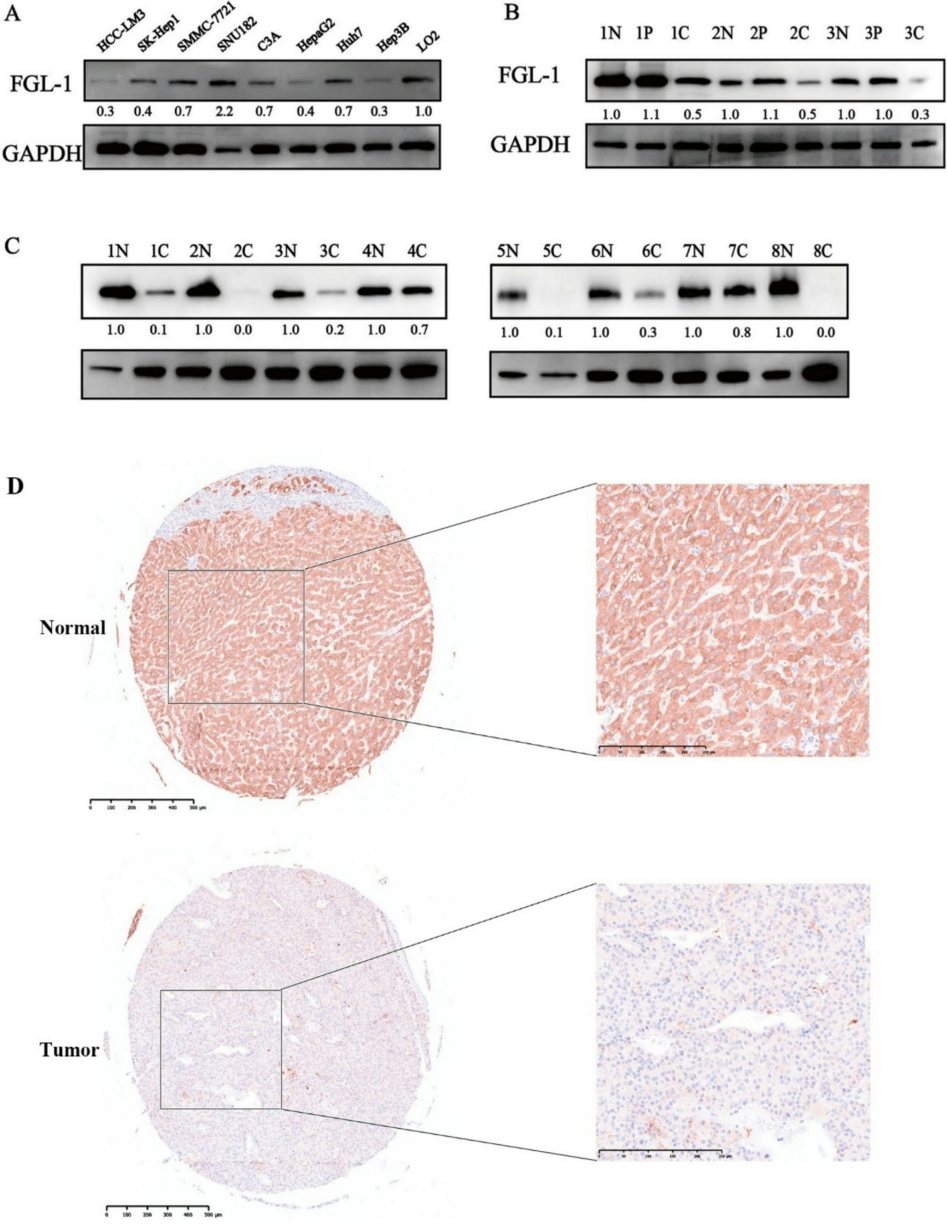
FGL1 expression showed obvious downregulation in several human HCC cell lines and HCC tissues. **A** Determination of FGL1 expression in several human HCC cell lines and the normal liver cell line via western blot analysis. **B** Determination of FGL1 expression with 3 pairs of HCC tissues and peri-tumor tissues and paired normal liver tissues via western blot analysis. **C** Determination of FGL1 expression with 8 pairs of HCC tissues and paired normal liver tissues via western blot analysis. **D** IHC staining for tumor tissues and adjacent normal liver tissues from HCC patients in the TMA. *N* normal liver tissue, *P* peri-tumor tissue, *C* cancer tissue; the numbers before N, *P* and C represent the group number

